# ANTI-CELL SENESCENT EFFECTS OF RAPAMYCIN AND THEIR ROLE IN DISEASES, INCLUDING ALZHEIMER’S

**DOI:** 10.1093/geroni/igz038.1352

**Published:** 2019-11-08

**Authors:** Viviana I Perez

**Affiliations:** Linus Pauling Institute, Oregon State University, Corvallis, Oregon, United States

## Abstract

Senescent cells contribute to age-related pathology and loss of function, and their selective removal improves physiology and extends longevity. We have shown that deficiency in Nrf2 in young Nrf2KO mice leads to an increase in senescent cells, with increased levels of pro-inflammatory cytokines in various tissues, including the brain. Both the cellular senescence and SASP decrease significantly when these mice are treated with rapamycin. Our current work focuses on determining whether cellular senescence contributes to premature AD-like pathogenesis in mouse models of AD. Indeed, it has been shown that Nrf2 deficiency in mouse models for AD leads to exacerbated pathology, suggesting that increased inflammation, due in part to the increased SASP-producing senescent cells, might be contribute to this phenotype. We are testing whether the burden of senescent cells in the brain on mouse models of AD can be reverted by rapamycin.

